# Immunization of Rabbits with a Quadrivalent *Shigella* Bioconjugate Vaccine Induces Functional Antibodies Reactive with *Shigella* Isolates from Kenya

**DOI:** 10.1128/msphere.01020-21

**Published:** 2022-05-25

**Authors:** Elizabeth A. Odundo, Hailey P. Weerts, Lillian Musila, Lilian Ogonda, Anita M. Dreyer, Joerg Schneider, Paula Carranza, Robert W. Kaminski

**Affiliations:** a Microbiology Hub Kericho, KEMRI-USAMRD-A/Kenya, Kericho, Kenya; b Department of Diarrheal Disease Research, Bacterial Diseases Branch, Walter Reed Army Institute of Research, Silver Spring, Maryland, USA; c Department of Biomedical Science and Technology, Maseno University, Maseno, Kenya; d LimmaTech Biologics AG, Schlieren, Switzerland; University of Florida

**Keywords:** *Shigella*, bioconjugate vaccine, preclinical, antibody, functional

## Abstract

Diarrheal diseases are a leading cause of global morbidity and mortality, disproportionately affecting children in resource-limited settings. Although improvements in hygiene and access to clean water are helpful, vaccines are considered essential due to the low infectious dose of *Shigella* species and increasing antibiotic resistance. Building on achievements with conjugate vaccines, a safe and immunogenic novel bioconjugate vaccine linking *Shigella* O-antigen to Pseudomonas aeruginosa exoprotein A has been developed to induce immunity against Shigella flexneri 2a, 3a, and 6 and S. sonnei. This study evaluated the breadth of reactivity and functionality of pooled serum from rabbits immunized with monovalent and quadrivalent *Shigella* bioconjugates formulated with or without an adjuvant against *Shigella* serotypes isolated in Kenya. Rabbit sera were assayed by colony blot for reactivity with 67 isolates of *Shigella* serotypes targeted by the vaccine, S. flexneri (2a, 3a, and 6) and S. sonnei, and 42 isolates of *Shigella* serotypes not targeted by the vaccine, S. flexneri (1b, 2b, 4a, and 4b), S. boydii, and S. dysenteriae. *Shigella* isolates testing positive in the colony blot assay were then used to assess functional activity using a bactericidal assay. Of the 41 *Shigella* isolates targeted by the vaccine, 22 were reactive with the adjuvanted quadrivalent and the respective monovalent rabbit sera. The S. flexneri 2a and 3a monovalent rabbit serum cross-reacted with S. flexneri 3a, 2b, and 2a, respectively. Immunization with the adjuvanted quadrivalent vaccine also induced cross-reactivity with isolates of S. flexneri 2b, 4a, and 4b. Collectively, these results suggest that the *Shigella* quadrivalent vaccine may be more broadly protective than designed, offering a promising solution to *Shigella* infections.

**IMPORTANCE** Diarrheal diseases are the third leading cause of death globally, disproportionally affecting low- to middle-income countries like Kenya, with *Shigella* species being the leading cause of bacterial diarrhea, especially in children. The low infectious dose and high antibiotic resistance levels complicate treatment, leading to long-term sequelae that necessitate control measures such as vaccines to reduce morbidity and mortality rates, especially among children under 5 years of age. A quadrivalent bioconjugate *Shigella* vaccine was recently developed to safely and effectively induce immunity against four important *Shigella* spp. This study demonstrates the breadth of reactivity and functionality of the parenterally administered bioconjugate vaccine by evaluating the ability of rabbit sera to bind and kill *Shigella* isolates recently collected in Kenya. These results suggest that the *Shigella* quadrivalent vaccine may be more broadly protective than designed and may offer a promising solution to the morbidity and mortality associated with *Shigella* infections.

## INTRODUCTION

Shigellosis, caused by *Shigella* spp., is a significant cause of bacterial diarrhea worldwide, accounting for approximately 165 million to 190 million cases and 1.1 million deaths per year, mainly in developing countries (1, 2). The low infective dose of 10 to 100 bacilli allows rapid and sustained transmission. Increasing antibiotic resistance further exacerbates treatment and management efforts. The growing global concern recognized by the World Health Organization (3) highlights the need for control measures such as environmental controls, proper hygiene, and vaccines to reduce the disease burden (4, 5).

Currently, no *Shigella* vaccine has been approved or licensed for widespread use. However, several promising *Shigella* vaccines are in the pipeline, with the leading candidates focused on conjugate vaccine development strategies (6, 7). The vaccines under development target the O-antigen from the most predominant *Shigella* serotypes, S. flexneri 2a, 3a, or 6 or S. sonnei (8), in single- or multivalent constructs. Building on previous achievements with conjugate *Shigella* vaccines (9), a bioconjugate comprised of *Shigella* O-antigen coupled to the carrier protein Pseudomonas aeruginosa exoprotein A (EPA) has been shown to be a safe, immunogenic, and tolerable vaccine (10, 11). Transitioning the monovalent S. flexneri 2a vaccine formulation from single valency to multivalency is required to induce immunity against the four major *Shigella* serotypes responsible for ~80% of global morbidity (10, 11). For more effective prevention and control, a broad-spectrum *Shigella* vaccine that can confer cross-protection against other virulent serotypes (S. flexneri 1b, S. flexneri 2b, S. dysenteriae, and S. boydii) (12) would be ideal.

Guinea pig and rabbit models have been successfully used to evaluate the immunogenicity of *Shigella* bioconjugate vaccines across monovalent and quadrivalent (4V) formulations and administered intramuscularly (i.m.) alone or in combination with an adjuvant. Although the antigen specificity and magnitude of the antibody response are critical parameters to evaluate, the ability of a vaccine to also induce functional antibodies is an important attribute that may differentiate protective from nonprotective immune responses. Therefore, in addition to immunoassays designed to assess antibody specificity and magnitude, a simple, high-throughput serum bactericidal assay (SBA) has been developed to assess the functionality of antibodies induced after infection or vaccination (13).

In this study, we evaluated the breadth of the antibody specificity from quadrivalent and monovalent bioconjugate-immunized rabbit serum against clinical *Shigella* isolates from Kenya representing the *Shigella* serotypes targeted (S. flexneri [2a, 3a, and 6] and S. sonnei) as well as related serotypes not targeted (S. flexneri 1b, 2b, 4b, and 4a; S. dysenteriae; and S. boydii) by the vaccine formulation. This study demonstrates the broad reactivity of the immune serum to serotypes beyond the targeted serotypes and the promising utility of the vaccine in the developing world.

## RESULTS

### Antibody responses in rabbits after immunization with monovalent and quadrivalent *Shigella* bioconjugate vaccines.

**FIG 1:**
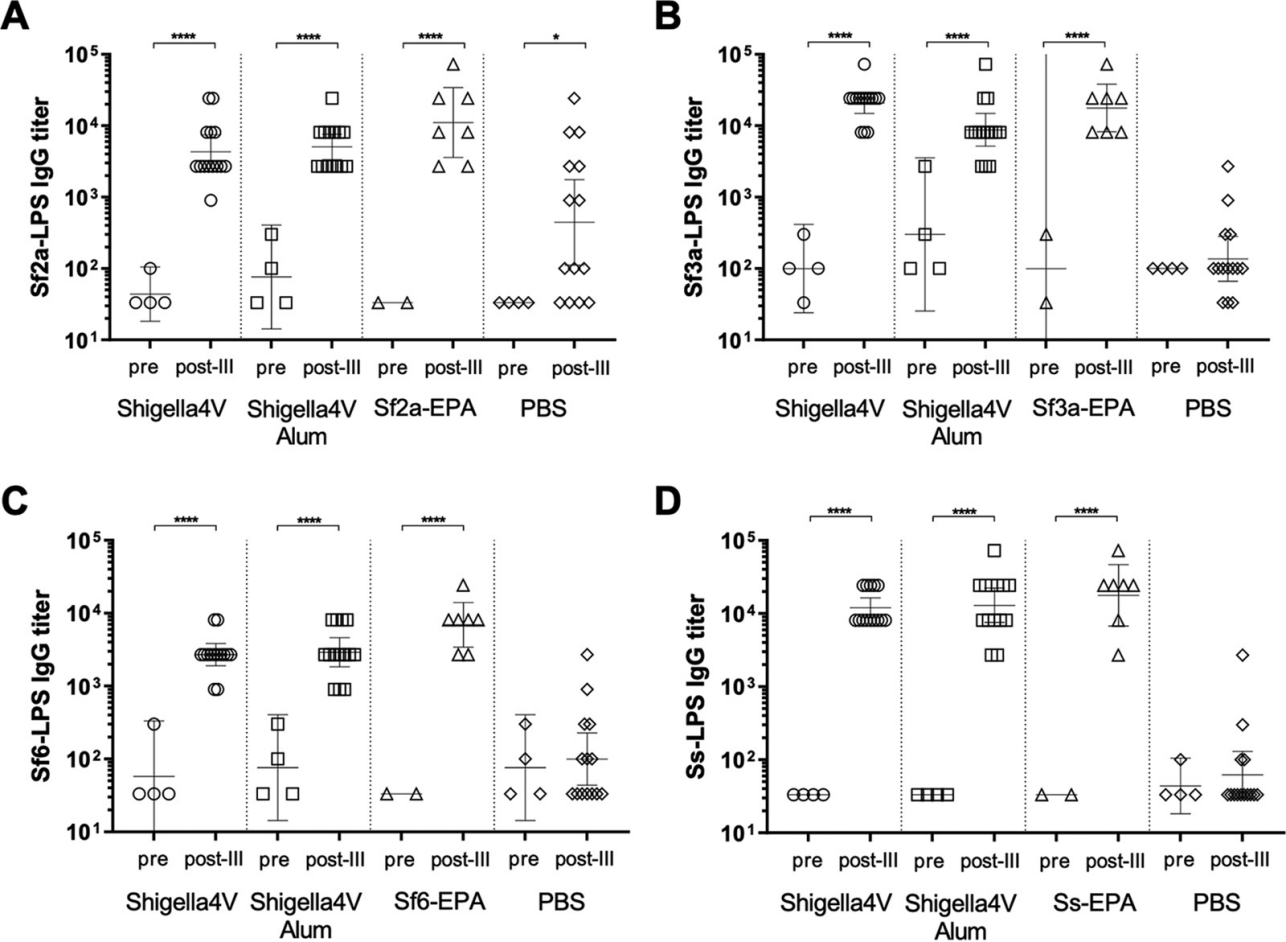
Sf2a-LPS-, Sf3a-LPS-, Sf6-LPS-, and S. sonnei LPS (Ss-LPS)-specific serum IgG titers in pre- and post-III immunization rabbit sera by treatment group. (A) Sf2a-LPS ELISA; (B) Sf3a-LPS ELISA; (C) Sf6-LPS ELISA; (D) Ss-LPS ELISA. Lines indicate the GMT ± the 95% confidence interval. ****, *P* < 0.0001; *, *P* < 0.05 (by one-way ANOVA).

Immunization of rabbits with the quadrivalent bioconjugate vaccine elicited IgG responses against lipopolysaccharide (LPS) purified from all four *Shigella* serotypes (Fig. 1). Post-III (2 weeks after the third injection) serum IgG titers against all four LPS antigens were significantly higher in rabbits vaccinated with 4V and the 4V vaccine with alum adjuvant (4V-Adj) than in preimmune sera or rabbits injected with phosphate-buffered saline (PBS) only (*P ≤ *0.0001). However, the 4V-Adj vaccine did not significantly enhance the magnitude of the LPS-specific IgG titers compared to the 4V titers (*P ≥ *0.2108), a result similar to the result achieved in the clinical setting with Flexyn2a (10).

The monovalent vaccines elicited strong anti-LPS IgG responses. The post-III serum IgG titers were significantly higher than those in the preimmune serum- or PBS-treated group (*P ≤ *0.0001). The LPS-specific IgG responses in the quadrivalent group were not significantly different from the IgG titers measured in the monovalent groups (*P ≥ *0.7735), indicating minimal interference with the multivalent formulation (Fig. 1).

In a fraction of preimmune serum pools (S. flexneri 2a LPS [Sf2a-LPS], Sf3a-LPS, and Sf6-LPS specific) and postimmunization PBS-treated and untreated rabbits, IgG titers were detectable, indicating that some rabbits had preexisting LPS-specific serum IgG. The post-III LPS-specific IgG titers of PBS-treated and untreated rabbits were not significantly different (*P ≥ *0.7932), except for Sf2a-LPS-specific IgG titers, which were higher in the PBS-treated animals (*P* = 0.0136). Serum from PBS-treated animals was screened for reactivity by colony blotting and bactericidal activity with historical *Shigella* isolates of all four *Shigella* serotypes and did not exhibit reactivity in either assay (data not shown). However, due to the reactivity in the enzyme-linked immunosorbent assay (ELISA), serum from PBS-treated rabbits was excluded from further analysis.

### *Shigella* serotype characterization and selection.

**FIG 2:**
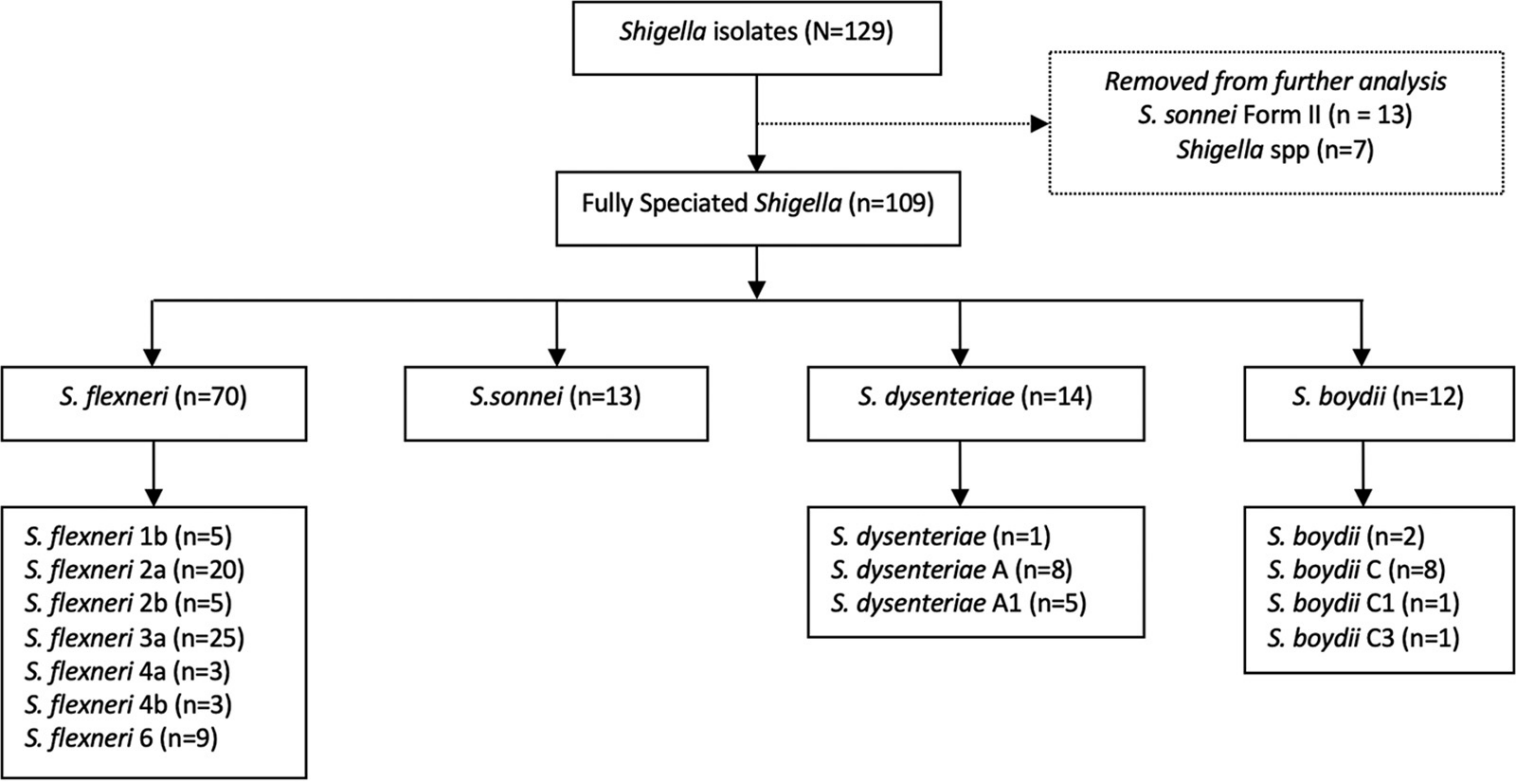
Flow chart of the distribution of *Shigella* serotypes verified by serotyping using set 2 Denka Seiken antisera containing 19 group and type antisera: 10 S. flexneri (group B), 3 S. sonnei (group D), 4 S. boydii (group C), and 2 S. dysenteriae (group A).

*Shigella* isolates were obtained from the Biobank of the Microbiology Hub Kericho (MHK) laboratory, and serotypes contained within the quadrivalent vaccine formulation, as well as *Shigella* serotypes not contained within the vaccine formulation, were selected. The selected 129 *Shigella* isolates subcultured on Trypticase soy blood agar (TSA) plates were verified (Fig. 2) to be S. flexneri (*n* = 70; 55%), S. sonnei (*n* = 13; 10%), S. dysenteriae (*n* = 14; 11%), and S. boydii (*n* = 12; 9%). Of the *Shigella* isolates that were serotyped, a total of 20 (S. sonnei form II [*n* = 13] and untypeable *Shigella* spp. [*n* = 7]) were not analyzed further.

### Results of Congo red binding assays.

Of the 129 isolates tested for virulence on Congo red (CR), 85% screened positive for CR uptake (CR positive [CR^+^]), with the following distribution: S. boydii (100%) and S. dysenteriae (100%), S. flexneri (96%), S. sonnei (42%), and *Shigella* spp (50%). As expected (14), most of the S. sonnei form II isolates (58%) did not retain their virulence and were Congo red negative (CR^−^). The distributions of *Shigella* serotypes selected for further analysis in colony spot blot assays and SBAs are summarized in Table 1. Relatively more S. flexneri 3a isolates were included in the analysis since the O-acetylation state can vary (15), which could impact potential reactivity with these isolates in colony blot analysis.

**TABLE 1:** *Shigella* spp. selected for analysis by colony blot and serum bactericidal assays

*Shigella* serotype(s)	Isolate abbreviation	No. of isolates per serotype	No. of CR^+^ isolates for colony blots	No. of isolates for SBA
*Shigella* serotypes contained within the vaccine formulation				
Shigella flexneri 2a	Sf2a	20	4	2
Shigella flexneri 3a	Sf3a	25	10	4
Shigella flexneri 6	Sf6	9	4	2
Shigella sonnei	SS-I	13	4	2

Subtotal		67	22	10


*Shigella* serotypes not within the vaccine formulation				
Shigella dysenteriae	SD	14	2	0
Shigella boydii C, C1, C3	SB/C2	12	3	0
Shigella flexneri 1b	Sf1b	5	5	0
Shigella flexneri 2b	Sf2b	5	4	2
Shigella flexneri 4a	Sf4a	3	2	2
Shigella flexneri 4b	Sf4b	3	3	1

Subtotal		42	19	5


Total		109	41	15

### Colony blot reactivity of rabbit serum antibodies after immunization with monovalent and quadrivalent *Shigella* bioconjugate vaccines with *Shigella* isolates from Kenya.

Paired rabbit serum samples collected before and after immunization with either monovalent or quadrivalent *Shigella* bioconjugate vaccines were utilized in colony blot assays to assess whether antibodies could bind to the surface-expressed bacterial antigens forming an antigen-antibody complex, visualizable by staining. All five *Shigella* control strains were incorporated into each colony blot. The positive-control strains were reactive with the serum samples collected after immunization with the monovalent and quadrivalent bioconjugate vaccines, while the negative control was non-reactive. As expected, no detectable reaction was observed with any of the rabbit sera collected pre-vaccination (data not shown).

Serum antibodies from rabbits immunized with 4V-Adj were reactive with 20/20 (100%) vaccine-targeted *Shigella* serotypes (Table 2). In contrast, serum antibodies from rabbits immunized with the 4V vaccine reacted with 14/20 (70%) of the vaccine-targeted *Shigella* isolates, which included all of the S. flexneri 3a and S. sonnei isolates but only 50% of the S. flexneri 2a isolates and none of the S. flexneri 6 isolates. As expected, reactivity was also observed with serum samples from rabbits immunized with monovalent bioconjugate vaccines but was mostly limited to the *Shigella* serotype specific for the O-antigen used in the vaccine formulation. However, there was limited cross-reactivity observed for sera from rabbits immunized with the S. flexneri 2a bioconjugate vaccine with one S. flexneri 3a isolate and one S. flexneri 2a isolate after immunization with either the monovalent S. flexneri 3a or S. flexneri 6 bioconjugate vaccine (Table 2).

**TABLE 2:** Reactivity of rabbit serum after immunization with monovalent or quadrivalent *Shigella* bioconjugate vaccines delivered without or with alum with *Shigella* isolates targeted by the vaccines^*a*^

*Shigella* serotype	Isolate ID	Postimmunization rabbit serum reactivity					
		Monovalent				Quadrivalent	
		S. flexneri 2a	S. flexneri 3a	S. flexneri 6	S. sonnei	4V	4V-Adj
S. flexneri 2a	SBA-K-S.f2a-003	+	−	−	−	−	+
	SBA-K-S.f2a-049*	+	+	−	−	+	+
	SBA-K-S.f2a-100*	+	−	−	−	−	+
	SBA-K-S.f2a-072	+	−	+	−	+	+

S. flexneri 3a	SBA-K-S.f3a-085*	−	+	−	−	+	+
	SBA-K-S.f3a-089*	−	+	−	−	+	+
	SBA-K-S.f3a-093	−	+	−	−	+	+
	SBA-K-S.f3a-098	−	+	−	−	+	+
	SBA-K-S.f3a-047	−	+	−	−	+	+
	SBA-K-S.f3a-001*	+	+	−	−	+	+
	SBA-K-S.f3a-040	−	+	−	−	+	+
	SBA-K-S.f3a-044*	−	+	−	−	+	+
	SBA-K-S.f3a-047	−	+	−	−	+	+
	SBA-K-S.f3a-111	−	+	−	−	+	+

S. flexneri 6	SBA-K-S.f6-008	−	−	+	−	−	+
	SBA-K-S.f6-050*	−	−	+	−	−	+
	SBA-K-S.f6-092	−	−	+	−	−	+
	SBA-K-S.f6-124*	−	−	+	−	−	+

S. sonnei	SBA-K-S.s1-105*	−	−	−	+	+	+
	SBA-K-S.s1-107*	−	−	−	+	+	+


Assay controls							
Positive	S. flexneri 2a 2457^T^	+	−	−	−	+	+
	S. flexneri 3a J17B	−	+	−	−	+	+
	S. flexneri 6, CCH060	−	−	+	−	+	+
	S. sonnei Moseley	−	−	−	+	+	+
Negative	K-Sspp-071	−	−	−	−	−	−

a An asterisk is used to indicate a *Shigella* isolate chosen for evaluation in the bactericidal assay. blank or −, no reactivity by colony blotting; + reactivity by colony blotting.

The reactivity of rabbit serum after immunization with either monovalent or quadrivalent *Shigella* bioconjugate vaccines was also tested against *Shigella* serotypes not targeted by the vaccine (Table 3). Cross-reactivity was observed with 7/19 (37%) of the *Shigella* isolates tested. Serum from rabbits immunized with 4V-Adj reacted with three (75%) of the S. flexneri 2b isolates, two (100%) of the S. flexneri 4a isolates, one (25%) of the S. flexneri 1b isolates, and one (33%) of the S. flexneri 4b isolates. Interestingly, animals immunized with the S. flexneri 2a monovalent bioconjugate had antibodies that cross-reacted with S. flexneri 2b in the colony blot assays. No cross-reactivity was observed for sera from monovalent S. flexneri 3a, S. flexneri 6, and S. sonnei bioconjugate-immunized rabbits with *Shigella* serotypes not targeted by the vaccine (Table 3), likely due to the different glycan compositions of the O-antigens.

**TABLE 3:** Reactivity of rabbit serum after immunization with monovalent or quadrivalent *Shigella* bioconjugate vaccines delivered without or with alum with *Shigella* isolates not targeted by the vaccines^*a*^

*Shigella* serotype	Isolate ID	Postimmunization rabbit serum reactivity					
		Monovalent				Quadrivalent	
		S. flexneri 2a	S. flexneri 3a	S. flexneri 6	S. sonnei	4V	4V-Adj
S. boydii	SBA-K-S.bC3-046	−	−	−	−	−	−
	SBA-K-S.bC-065	−	−	−	−	−	−
	SBA-K-S.bC-095	−	−	−	−	−	−

S. dysenteriae	SBA-K-S.dA-012	−	−	−	−	−	−
	SBA-K-S.dA1-041	−	−	−	−	−	−

S. flexneri 2b	SBA-K-S.f2b-066	−	−	−	−	−	−
	SBA-K-S.f2b-131*	+	−	−	−	−	+
	SBA-K-S.f2b-132*	+	−	−	−	−	+
	SBA-K-S.f2b-133*	+	−	−	−	−	+

S. flexneri 1b	SBA-K-S.f1b-021^*b*^	−	−	−	−	+	+
	SBA-K-S.f1b-025	−	−	−	−	−	−
	SBA-K-S.f1b-042	−	−	−	−	−	−
	SBA-K-S.f1b-102	−	−	−	−	−	−
	SBA-K-S.f1b-103	−	−	−	−	−	−

S. flexneri 4a	SBA-K-S.f4a-032*	−	−	−	−	−	+
	SBA-K-S.f4a-039*	−	−	−	−	−	+

S. flexneri 4b	SBA-K-S.f4b-068*	−	−	−	−	−	+
	SBA-K-S.f4b-090	−	−	−	−	−	−
	SBA-K-S.f4b-115	−	−	−	−	−	−


Assay controls							
Positive	S. flexneri 2a 2457^T^	+	−	−	−	+	+
	S. flexneri 3a J17B	−	+	−	−	+	+
	S. flexneri 6 CCH060	−	−	+	−	+	+
	S. sonnei Moseley	−	−	−	+	+	+
Negative	K-Sspp-071	−	−	−	−	−	−

a An asterisk is used to indicate a *Shigella* isolate chosen for evaluation in the bactericidal assay. blank or −, no reactivity by colony blotting; +, reactivity by colony blotting.

b Sample SBA-K-S.f1b-021 was reactive by colony blotting but was not utilized for bactericidal analysis.

### Functional activity of serum antibodies induced after immunization with monovalent or quadrivalent *Shigella* bioconjugate vaccines.

The rabbit serum samples reactive in the colony blot assays were assessed for bactericidal activity (Table 4). All *Shigella* isolates showed optimal growth in the SBA after 16 to 18 h of incubation at 29°C and 26°C for S. sonnei, resulting in microcolony growth sufficient to be detected. Growth conditions were similar to those for historical *Shigella* strains (S. flexneri 2a strain 2457^T^, S. sonnei 53G and Moseley, S. flexneri 6 CCH060, and S. flexneri 3a J17B), as previously reported (13), with a modification for S. flexneri 6 CCH060, which was incubated at 29°C instead of 26°C. The 50% cutoff value established in this assay is standard in similar studies (13).

**TABLE 4:** Bactericidal activity of rabbit antibodies induced after immunization with monovalent or quadrivalent *Shigella* bioconjugate vaccines formulated with or without alum against selected *Shigella* strains isolated in Kenya and historical laboratory strains^*b*^

*Shigella* serotype	Isolate ID	Fold increase^*a*^ in antibodies induced in rabbits after immunization with:					
		Monovalent				Quadrivalent	
		S. flexneri 2a	S. flexneri 3a	S. flexneri 6	S. sonnei	4V	4V-Adj
*Shigella* serotypes contained within the vaccine formulation							
S. flexneri 2a	SBA-K-S.f2a-049	70	19	—	—	15	12
	SBA-K-S.f2a-100	111	—	—	—	—	24
	SBA-K-S.f2a-072	100	—	36	—	9	9
S. flexneri 3a	SBA-K-S.f3a-001	2	7	—	—	4	32
	SBA-K-S.f3a-044	—	49	—	—	34	34
	SBA-K-S.f3a-085	—	40	—	—	54	20
	SBA-K-S.f3a-089	—	98	—	—	37	42
S. flexneri 6	SBA-K-S.f6-050	—	—	46	—	—	70
	SBA-K-S.f6-124	—	—	54	—	—	113
S. sonnei	SBA-K-S.s1-105	—	—	—	746	68	274
	SBA-K-S.s1-107	—	—	—	663	74	68

*Shigella* serotypes not within the vaccine formulation							
S. flexneri 2b	SBA-K-S.f2b-131	67	—	—	—	—	41
S. flexneri 2b	SBA-K-S.f2b-132	62	—	—	—	—	25
S. flexneri 4a	SBA-K-S.f4a-032	—	—	—	—	—	7
S. flexneri 4a	SBA-K-S.f4a-039	—	—	—	—	—	7
S. flexneri 4b	SBA-K-S.f4b-068	—	—	—	—	—	9

Historical laboratory strains							
S. flexneri 2a	2457^T^	117	40	11	6	7	18
S. flexneri 3a	J17B	2	34	1	2	47	15
S. flexneri 6	CCH060	3	13	14	4	506	188
S. sonnei	Moseley	5	1	1	11,918	2,737	3,064

a Fold increase calculated by dividing the postimmunization (post-III or day 42 pooled rabbit serum) bactericidal titer by the preimmunization (pre or day 0 pooled rabbit serum) bacterial titer.

b Responders (shaded) were defined as having a ≥8-fold increase in bactericidal titers over the baseline. — indicates that the sample was not tested for bactericidal activity because the isolate was unreactive by colony blotting.

Consistent with the colony blot results, bactericidal activity was low to undetectable in pre-immunization pooled rabbit serum (data not shown). Sera from rabbits immunized with the monovalent S. flexneri 2a bioconjugate were capable of killing the three S. flexneri 2a isolates as well as the two S. flexneri 2b isolates (Table 4). Similarly, sera from animals immunized with the monovalent S. flexneri 3a or S. flexneri 6 bioconjugate vaccine were capable of killing homologous *Shigella* serotypes but also exhibited the ability to kill isolates of other heterotypic serotypes. In contrast, serum from rabbits immunized with the monovalent S. sonnei bioconjugate had bactericidal activity against S. sonnei isolates but no cross-reactivity with other serotypes.

Serum from rabbits immunized with the quadrivalent *Shigella* bioconjugate (4V) had bactericidal activity against two of the S. flexneri 2a strains, two of the S. sonnei isolates, and all four of the S. flexneri 3a isolates but none of the S. flexneri 6 isolates. In stark contrast, serum from rabbits immunized with 4V-Adj had bactericidal activity against all 16 *Shigella* isolates representing the four *Shigella* serotypes targeted by the vaccine formulation, in addition to three *Shigella* serotypes not specifically targeted by the vaccine (Table 4). The magnitudes of the bactericidal titers induced after immunization with the monovalent and quadrivalent bioconjugate vaccines varied widely. The highest level of reactivity was noted with the S. sonnei antisera, and the lowest was noted with the S. flexneri 6 monovalent antisera.

A correlation analysis was performed to assess the relationship between the SBA titers and the LPS-specific serum IgG ELISA titers (Table 5). SBA and LPS-specific ELISA titers were significantly correlated for all serotypes investigated (S. flexneri 2a, 3a, and 6 and S. sonnei), with the strongest correlations being observed with S. sonnei (Pearson *r* ≥ 0.998; *P* < 0.0001) (Table 5)- and S. flexneri 3a (Pearson *r* = 0.961; *P* < 0.0001) (Table 5)-immunized rabbits.

**TABLE 5:** Correlation of *Shigella* LPS-specific serum IgG ELISA titers and serum bactericidal titers^*a*^

Parameter	Value			
	S. flexneri 2a	S. flexneri 3a	S. flexneri 6	S. sonnei
Pearson *r*	0.909	0.961	0.701	0.998
95% confidence interval	0.654–0.979	0.838–0.991	0.127–0.923	0.990–0.999
*P* value	0.0003	<0.0001	0.024	<0.0001

a Titers were log transformed, and a Pearson correlation was performed. SBA titers from pooled serum samples were compared to geometric mean ELISA titers from individual serum samples.

## DISCUSSION

*Shigella* spp. are one of the leading global causes of diarrheal morbidity and mortality, posing a serious public health challenge among children in resource-limited settings (16). Certain *Shigella* spp., when coupled with multidrug resistance, can cause severe disease, resulting in long-lived sequelae such as toxic megacolon, irritable bowel syndrome, hemolytic-uremic syndrome, reactive arthritis, and stunted physical and cognitive growth in children amid a myriad of other diarrheal symptoms (6, 17).

Data from the Global Enteric Multicenter Study (GEMS) indicate that S. sonnei and S. flexneri 3a, 2a, and 6 are the predominant serotypes responsible for approximately 80% of *Shigella* diarrheal infections globally, resulting in the need for a broadly reactive vaccine. Generally, for conjugate vaccine approaches targeting the O-antigen of *Shigella*, this would translate into a quadrivalent *Shigella* vaccine (10, 12). In addition to the breadth of coverage, the magnitude and functionality of the immune response will likely define an effective vaccine approach, especially in children under the age of 5 years.

Vaccination remains the cornerstone for global reduction of mortalities, especially childhood disease prevention, and improved quality of life across all ages. Although advances have been made with several *Shigella* vaccines, many challenges remain. Pathogen diversity, vaccine safety, efficacy, immunogenicity, the lack of clear correlates of protection or predictive animal models, the availability and accessibility of the target population, the lack of population confidence, and vaccine affordability are still being addressed (18, 19). However, significant progress has been made with *Shigella* serotype-specific O-antigen conjugate vaccines (20) and, recently, with bioconjugate technology that appears to offer a more intrinsic advantage (10, 14). The *Shigella* bioconjugate approach has been tested in two clinical studies (10, 21, 22) using the monovalent Flexyn2a (S. flexneri 2a O-antigen conjugated to the carrier protein EPA) vaccine and was shown to be well tolerated, immunogenic, and efficacious against the most severe shigellosis. The specific and functional anti-S. flexneri 2a antibody responses observed in these clinical studies (10, 11) agree with the rabbit serum results following serotype-specific monovalent bioconjugate vaccination against the specific *Shigella* strains (Table 2) in this study. The results from these first clinical studies encouraged the development of a quadrivalent bioconjugate vaccine (*Shigella* 4V) targeting S. sonnei and S. flexneri 2a, 3a, and 6, which is now being evaluated in a phase 1/2a age-descending study in Kenya.

Before clinical evaluation, the *Shigella* 4V vaccine was tested in rabbits and guinea pigs to evaluate immunogenicity, immune competition, and the generation of functional antibody responses. The data presented here indicate that the 4V vaccine was capable of inducing functional antibody responses to the four *Shigella* serotypes targeted by the quadrivalent vaccine albeit at levels comparable to or lower than those of the functional responses induced with the monovalent *Shigella* vaccines. However, the addition of alum to the 4V vaccine formulation not only enhanced the magnitude of the functional response but also increased the breadth of cross-reactivity to *Shigella* serotypes not targeted by the vaccine. The combination of bacterial binding in a colony blot format and bactericidal activity after immunization with the 4V vaccine and adjuvant formulation holds the promise of a vaccine with broader coverage.

In this study, the four major *Shigella* serotypes targeted by the quadrivalent bioconjugate vaccine formulation and other *Shigella* serotypes of global importance were utilized to understand the breadth of the functional antibody response induced after vaccination. These additional *Shigella* serotypes included S. dysenteriae, responsible for epidemics and outbreaks after natural disasters or infrastructure breakdowns (23); S. boydii; S. flexneri 1b; S. flexneri 2b; S. flexneri 4a (12, 24); and S. flexneri 4b. Although S. flexneri 7a was previously identified in Kenya (12), this serotype was not included due to the unavailability of antisera used for serotype verification by slide agglutination. However, the isolates tested cover a broader geographical region and broader age groups (data not shown) in Kenya than in previous studies (12, 24). Similar to a previous study (14), most of the *Shigella* isolates screened for use in this study retained their virulence plasmid, with 85% testing positive and binding Congo red.

Cross-protection among Shigella flexneri serotypes has been explored previously (15, 25–27) in the context of animal studies and clinical evaluations. Emphasis has been placed on the ability of a vaccine to confer a significant degree of protection against the most common *Shigella* serotypes, especially those with shared antigenic structures {group B [serotypes 3/4, 6, and 7(8)]} (15). Immunization of guinea pigs with a bivalent S. flexneri 2a/3a vaccine significantly protected the vaccinated guinea pigs against challenge with S. flexneri Y, 1b, 2b, and 5b. Still, it offered nominal protection against S. flexneri 1a, 4b, and 6 (15). Similarly, in this study, rabbits immunized with the bioconjugate S. flexneri 2a monovalent vaccine had cross-reactive antibodies with S. flexneri 2b (Table 4). Immunization with the S. flexneri 3a monovalent vaccine induced cross-reactive antibodies to one of three S. flexneri 2a isolates. Similarly, serum from animals immunized with the monovalent S. flexneri 6 bioconjugate also killed one of the S. flexneri 2a isolates. There was no significant cross-reactivity of serum from animals immunized with S. sonnei monovalent vaccine toward other *Shigella* serotypes nor from *S. flexneri* 2a, 3a, or 6 sera toward *S. sonnei* strains; however, this O-antigen does not share structural similarities with the other serotypes tested. Interestingly, immunization with the 4V-Adj and 4V bioconjugate showed broader cross-reactivity with the prevalent serotypes than vaccination with the monovalent vaccines and additional serotypes within group B (S. flexneri 1b, 2b, 4a, and 4b). These findings support the assumption that a higher degree of cross-protection can be achieved by combining *Shigella* antigenic and group factors within a vaccine (15).

Evaluating the correlation between the development of serotype-specific antibody responses (as measured by an LPS-specific ELISA) and the functional activity of those antibodies (as measured by an SBA) may give additional insights into the development of protective immune responses. Identifying the antigen specificity of the antibodies responsible for bactericidal activity can help determine specific antigenic targets that could be important in the design of protective vaccines. A significant correlation was found between LPS-specific serum IgG ELISA titers and bactericidal activity across all historical *Shigella* strains evaluated (Table 5), indicating that LPS-specific antibodies likely contribute to protective immunity. Although still significant, lower *r* values were observed in the S. flexneri 6-immunized groups than in the groups immunized against the other *Shigella* serotypes. Bactericidal antibodies specific for other antigenic targets (such as IpaB or IpaC) may also be important contributors to protection. Additionally, other antibody isotypes, such as IgM, may also contribute to killing. Further investigations into the specificity and isotype of the bactericidal antibodies may help explain why the correlation was less robust for S. flexneri 6. While the information presented here clearly demonstrates a role for LPS-specific IgG in bactericidal activity, future studies into other antigenic targets and antibody isotypes are warranted to fully characterize the functional immune response induced by these vaccines (16).

Although the mechanisms of action afforded by vaccine adjuvants are mostly unknown, the effects of adjuvants on vaccine-induced immune responses can be multifactorial. For example, the inclusion of an adjuvant (such as alum) in a vaccine formulation can augment the magnitude of the immune response, redirect the phenotype of the immune response, or expand the breadth of the immune response, as shown with proteins (20, 28). Therefore, adjuvants may be useful in vaccine development to reduce the amount of vaccine required to reach an immunological threshold, in terms of either the vaccine dose amount or the number of vaccinations (dose sparing), or to enhance the immunogenicity and safety of a vaccine in populations where immune responses are not as robust, such as in small children or infants (3, 28). The addition of alum to the *Shigella* 4V bioconjugate vaccine formulation increased the breadth of the immune response cross-reactivity in the colony blot assays and SBAs (Tables 2 to 4). Previous clinical studies with a monovalent Flexyn2a bioconjugate (10) did not detect an increase in the magnitude or a change in the phenotype of the immune response induced, but those studies did not directly measure any changes in the breadth of the immune response in terms of cross-reactivity with other *Shigella* serotypes. Archived samples from those studies could be utilized to investigate these hypotheses using a study structure similar to the one implemented with the rabbit serum. Nevertheless, these results demonstrate an added advantage of alum to the quality of the immune response induced after immunization with the quadrivalent formulation and suggest that the vaccine could protect against more than the four *Shigella* serotypes targeted by the vaccine. If clinical evaluations, which are under way in Kenya, demonstrate a more broadly protective immune response, the *Shigella* bioconjugate may offer a viable solution to the morbidity and mortality associated with *Shigella* infections. As vaccine development efforts progress, the multivalent *Shigella* vaccines’ ability to elicit broadly cross-reactive immunity should be explored, especially against globally predominant *Shigella* serotypes.

## MATERIALS AND METHODS

### Ethical considerations. (i) Animal care and use.

All of the experimentation involving animals was done under the frame of ethical protocol CE/Sante/E/001 (immunization and production of sera/polyclonal antibodies) approved by the ethical committee of CER Groupe (agreement number LA1800104). Agreement LA1800104 was bestowed by the Federal Public Service of the Walloon Region (Belgium). The experimentation was performed according to legislation in force at the moment of the studies, thus following the guidelines established at the European level (Directive 2010/63/EU revising Directive 86/609/EEC on the protection of animals used for scientific purposes), the Belgian level (Arrêté Royal Relatif à la Protection des Animaux d’Expérience, AR 2013/05/29), and the regional level (Code Wallon du Bien-Être Animal 03/10/2018). We adhered to the policies for protection of human subjects as prescribed in Army Regulation 70 -25.

CER Groupe is compliant with all regulations and guidelines for the care, welfare, and ethical treatment of animals and, as a minimum, with the following core principles: access to species-appropriate food and water; access to species-specific housing, including species-appropriate temperature and humidity levels; access to humane care and a program of veterinary care; the ability to demonstrate species-specific behavior; adherence to 3R principles (Replacement, Reduction and Refinement) in the design of *in vivo* studies; study design reviewed by an institutional ethical review panel; commitment to minimizing pain and distress during *in vivo* studies; and work performed by appropriately trained staff.

### (ii) Use of pooled serum and *Shigella* isolates.

Permission to use the pooled serum samples in Kenya was granted by LimmaTech Biologics AG through the Walter Reed Army Institute of Research (WRAIR) Subunit Enteric Vaccines and Immunology (SEVI) department. The approval to conduct the study using *Shigella* isolates from Kenya was granted by the institutional review board (IRB) of KEMRI (KEMRI/SERU/CCR/125/3900) and given an exemption determination by WRAIR as nonhuman research.

### Rabbit immunizations.

New Zealand White rabbits (*n* = 14/quadrivalent group; *n* = 7/monovalent group) were intramuscularly (i.m.) vaccinated three times at 2-week intervals (days 0, 14, and 28) with 0.5 mL containing either monovalent or quadrivalent *Shigella* bioconjugate (*Shigella* 4V) products targeting S. flexneri 2a, 3a, and 6 and S. sonnei (29, 30). The vaccines contained a 1-μg polysaccharide (PS) dose of each *Shigella* serotype being targeted; the monovalent vaccine received 1 μg of the specific serotype, and the quadrivalent vaccine received 1 μg of each serotype (total of 4 μg of glycan). The quadrivalent vaccine was delivered with and without alum as an adjuvant. Serum antibody responses directed to LPS from the four *Shigella* serotypes and the carrier protein, exoprotein from Pseudomonas aeruginosa (EPA), were monitored prior to immunization and 2 weeks after the third injection (pre- and post-III, respectively) by an ELISA (EPA and untreated animal data are not shown). Control groups consisted of animals vaccinated i.m. with phosphate-buffered saline (PBS) (control) and a null treatment group.

### Determination of LPS-specific serum IgG titers by an ELISA.

Microtiter 96-well plates (MaxiSorp, Nunc; Thermo Scientific) were coated with 100 μL per well of *Shigella* LPS (5 μg/mL) and methylated bovine serum albumin (BSA) (10 μg/mL) in PBS. After incubation overnight at 4°C, the plates were washed with PBS–0.05% Tween 20 (PBS-T) and incubated for 2 h with 300 μL of PBS–5% skimmed milk powder. Serial 3-fold dilutions (in PBS) of each test serum sample in duplicate were incubated on a shaker for 1 h at room temperature. After washing in PBS-T, the plates were incubated with peroxidase-conjugated goat anti-rabbit IgG (Fc) antibodies (catalog number 111-035-008; Jackson ImmunoResearch) diluted 1:100,000 on a shaker for 1 h at room temperature. Plates were washed in PBS-T, a tetramethylbenzidine (TMB) substrate solution (catalog number T4444; Sigma) was added to each well (100 μL/well), and the plates were incubated for 6 min. The reaction was stopped by the addition of 100 μL of 1 N sulfuric acid (H_2_SO_4_), and the optical density (OD) was read at 450 nm. The individual endpoint titers were determined as the highest dilution above the mean OD value plus 3 standard deviations (SD) of the buffer-only controls or 0.02 when the mean OD value plus 3 SD was <0.02. Responders were defined as those samples with a ≥4-fold titer increase in post- versus preimmunization rabbit sera. One-way analysis of variance (ANOVA) was used to determine the significance of the LPS IgG titers between the pre- and post-III immunized rabbits by treatment group.

### *Shigella* strains and serum samples.

The deidentified *Shigella* strains used in this study were isolated from diarrheal stool specimens of participants enrolled between 2010 and 2019 in an ongoing approved outpatient hospital-based case-control study (protocol number KEMRI SERU 1549/WRAIR 1549). The isolates were stored in 50% glycerol at −80°C in the Microbiology Hub Kericho (MHK). The pre- and post-III bioconjugate-vaccinated pooled rabbit sera (the 4-valent vaccine with alum [4V-Adj] or without alum [4V] and the monovalent vaccine) were provided by LimmaTech Biologics (LMTB), and *Shigella* positive-control strains (S. flexneri 2a strain 2457^T^, S. flexneri 3a J17B, S. flexneri 6 CCH060, and S. sonnei Moseley) used in the study were obtained from WRAIR. A Congo red (CR)-negative *Shigella* isolate, K-Sspp-071 (provided by MHK), was used as a negative control.

### *Shigella* species serotype verification.

A total of 129 *Shigella* isolates were systematically selected from the MHK Biobank. The isolates were subcultured on Trypticase soy blood agar (TSA) plates (Becton, Dickinson, USA), incubated overnight at 37°C, and checked for pure isolated colonies. The *Shigella* groups and types were verified by slide agglutination using commercial antiserum set 2 (Denka Seiken Co. Ltd., Tokyo, Japan) according to the manufacturer’s instructions.

### Screening of *Shigella* colonies.

The *Shigella* isolates were streaked onto CR solid medium and incubated for 16 to 18 h at 39°C. A subset of the CR-positive isolates was randomly chosen for colony blot analysis, representing *Shigella* serotypes targeted by the vaccine formulation (S. flexneri 2a, 3a, and 6 and S. sonnei) as well as serotypes not specifically targeted by the vaccine (Table 1). One CR-negative isolate (K-Sspp-071) was selected as a negative control.

### Colony blotting: reactivity of rabbit sera with the virulent *Shigella* isolates.

Prelabeled nitrocellulose disk membranes (Bio-Rad, USA) were layered onto TSA plates and inoculated with 2 μL of a virulent *Shigella* isolate or a control strain. Following incubation overnight at 37°C, the membranes were transferred into a container and blocked on an orbital shaker in 2% casein buffer for 30 min at 23°C ± 2°C. The membranes were washed four times for 15 min each in Tris-buffered saline (TBS) and wash buffer (TBS plus 0.05% Triton X-100) and incubated in the respective quadrivalent and monovalent bioconjugate-vaccinated rabbit serum (primary antibody) diluted 1:250 in 2% casein buffer. After a 2-h incubation at 23°C ± 2°C, the membranes were washed, transferred into protein A-alkaline phosphatase (Sigma) at 2 μg/mL for 1 h, and washed; color was developed in a fast red-naphthol AS-TR substrate (Sigma) solution for 30 min; and the membranes were dried at 23°C ± 2°C. The spots on the colony blots were compared to the positive- and negative-control colors to determine positivity (reactive) or negativity (nonreactive). A subset of reactive *Shigella* serotypes was selected for evaluation in functional antibody assays.

### Serum bactericidal assay.

The bactericidal activities of serum samples against historical strains (S. flexneri 2a strain 2457^T^, S. sonnei 53G and Moseley, S. flexneri 6 CCH060, and S. flexneri 3a J17B) were assessed as previously described (13, 31). Briefly, serum samples from rabbits immunized with quadrivalent and monovalent bioconjugate vaccines were diluted 1:10 with serum bactericidal assay (SBA) buffer and heat inactivated at 56°C for 30 min. The serum samples were assayed for bactericidal activity against 10 *Shigella* isolates representing S. sonnei and S. flexneri 2a, 3a, and 6 serotypes contained within the LMTB quadrivalent (4V) bioconjugate vaccine used for rabbit immunization. Additionally, six *Shigella* isolates representing other serotypes of S. flexneri (S. flexneri 1b, 2b, 4a, and 4b) were assayed. Prior to conducting the SBA, each isolate was tested to determine the optimal growth times and temperatures to yield acceptable colony counts and colony sizes for the assay. Colony counts were enumerated using NIST Integrated Colony Enumerator (NICE) software, and an Excel-based software program, Opsotiter, was used to determine the killing index (KI) or bactericidal titer of the serum (32). The KI was defined as the inverse of the dilution of serum that kills ≥50% of bacteria. An interpolated titer was determined using an algorithm, described by the following formula, where log-transformed serum dilutions are analyzed to determine the 50% KI:

KI (X50)=10[log⁡ X1 + (Y50 − Y1) × (log⁡ X2  −log⁡ X1)(Y2 − Y1)]

The results are then converted back on a normal arithmetic scale, and the interpolated bactericidal titer is reported. The fold increase between the pre- and postbactericidal titers was calculated, and fold increases of ≥4 were considered responders.

A Pearson correlation analysis was performed for each serotype to assess the relationship between the SBA titers and the LPS-specific serum IgG ELISA titers from the rabbit serum. Monovalent- and quadrivalent-vaccinated groups were included in the analysis, and comparisons were conducted across homologous serotype-specific data sets (i.e., S. flexneri 2a SBA versus S. flexneri 2a LPS-specific serum IgG). Log-transformed LPS-specific serum IgG ELISA geometric mean titers (GMTs) were compared to log-transformed SBA titers using Prism version 8.0 (alpha = 0.05).
